# Elastic intramedullary nailing and DBM-Bone marrow injection for the treatment of simple bone cysts

**DOI:** 10.1186/1477-7819-5-111

**Published:** 2007-10-04

**Authors:** Anastasios D Kanellopoulos, Andreas F Mavrogenis, Panayiotis J Papagelopoulos, Panayotis N Soucacos

**Affiliations:** 1 First Department of Orthopaedics, Athens University Medical School, Athens Greece

## Abstract

**Background:**

Simple or unicameral bone cysts are common benign fluid-filled lesions usually located at the long bones of children before skeletal maturity.

**Methods:**

We performed demineralized bone matrix and iliac crest bone marrow injection combined with elastic intramedullary nailing for the treatment of simple bone cysts in long bones of 9 children with a mean age of 12.6 years (range, 4 to 15 years).

**Results:**

Two of the 9 patients presented with a pathological fracture. Three patients had been referred after the failure of previous treatments. Four patients had large lesions with impending pathological fractures that interfered with daily living activities. We employed a ratio to ascertain the severity of the lesion. The extent of the lesion on the longitudinal axis was divided with the normal expected diameter of the long bone at the site of the lesion. The mean follow-up was 77 months (range, 5 to 8 years). All patients were pain free and had full range of motion of the adjacent joints at 6 weeks postoperatively. Review radiographs showed that all 7 cysts had consolidated completely (Neer stage I) and 2 cysts had consolidated partially (Neer stage II). Until the latest examination there was no evidence of fracture or re-fracture.

**Conclusion:**

Elastic intramedullary nailing has the twofold benefits of continuous cyst decompression, and early immediate stability to the involved bone segment, which permits early mobilization and return to the normal activities of the pre-teen patients.

## Background

Simple or unicameral bone cysts are common benign fluid-filled lesions usually located at the long bones of children before skeletal maturity. They form 3% of all bone lesions in this age group. A pathological fracture is the presenting symptom in most patients [1,2].

The etiology and pathogenesis of simple bone cysts is uncertain. Cysts close to the growth plate show biological activity, are usually expansile, and recur more often than those away from the growth plate [3-5].

Various treatment options have been reported for simple bone cysts including crushing of the cyst wall and onlay grafting [6], total resection with bone grafting [7,8], subtotal resection with and without bone grafting [9,10], curettage combined with bone grafting [3], allografting with freeze-dried crushed cortical bone [11], homologous cancellous bone chips [12], high-porosity hydroxyapatite components [13] or plaster-of-Paris pellets [14] and cryosurgery [15], injection of methylprednisolone [16-18], bone marrow or bone substitutes [19-24], decompression with drilling or screws [25,26], and intramedullary nailing [27-30].

To our knowledge, combined biological and mechanical treatment of simple bone cysts has not been previously reported. The purpose of this study is to describe the technique and present the results of demineralized bone matrix and autologous bone marrow injection in addition to intramedullary cannulation and stabilization for the treatment of large and active simple bone cysts.

## Patients and Methods

Nine children that were admitted at the authors' institution for a unicameral bone cyst between 1999 and 2002 met the criteria above. There were 7 boys and 2 girls with a mean age of 12.6 years (range, 4 to 15 years) at the time of surgery. These patients have been followed until currently for the purpose of this study.

**Figure 1:**
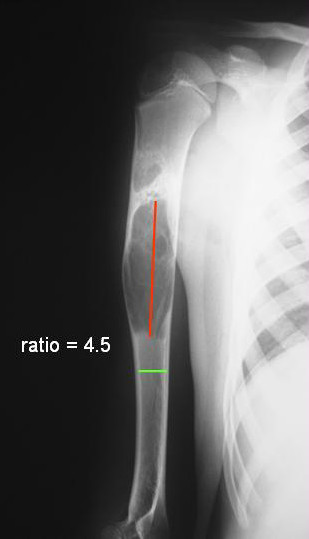
We arbitrarily employed a ratio to ascertain the severity of a simple bone cyst. The extent of the lesion on the longitudinal axis was divided with the normal expected diameter of the long bone at the site of the lesion. The presented in this study method of treatment has been performed in large unicameral bone cysts that occupied more than 2 times the physiologic diameter of the long bone at the site of the lesion.

The diagnosis was based on typical imaging, cystographic features and histology. Previous studies have used the cyst index [31] and the cyst diameter [32] to measure the state and progress of cystic activity. We employed a radiographic ratio to ascertain the severity of the lesion. The extent of the lesion on the longitudinal axis was divided with the normal expected diameter of the long bone at the site of the lesion (Figure 1). The presented herein method of treatment has been performed in large simple bone cysts that occupied more than 2 times the physiologic diameter of the long bone at the site of the lesion.

All patients had large and active metaphyseal lesions adjacent to the physis of a long bone. Six cysts were located at the proximal humerus and three cysts were located at the proximal femur.

Two of the 9 patients had been referred with a pathological fracture of the femur and the proximal humerus. In these patients, diagnosis was obtained using imaging techniques including standard radiographs, computed tomography scans and magnetic resonance imaging. The fractures had been initially treated non-operatively with closed reduction and cast immobilization. Treatment of the cysts initiated 6 weeks after the occurrence of the fractures, to prevent recurrence, and to provide stability to the bone and early mobilization of the patients. Three patients had been referred after failure of previous treatments. One patient had a proximal femoral cyst that persisted after two open grafting operations using autogenous and allogenic bone. Two patients with a proximal femoral and humeral cyst had been referred after failed repeated steroid injections. The remaining four patients had large lesions with impending pathological fracture that interfered with daily living activities.

### Operative technique

**Figure 2:**
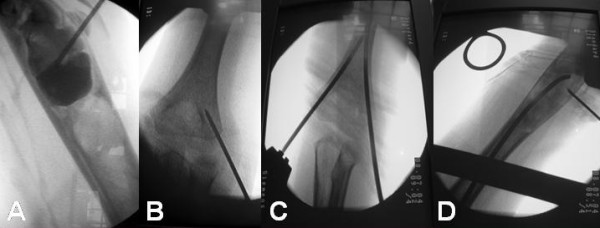
**(A) **A cystogram was performed and tissue was obtained for histological examination. **(B) **Under image-intensifier control, kirschner wires were drilled in appropriate positions on both medial and lateral cortices. **(C) **The entry holes of the nails were created with cannulated drills over the kirschner wires, and the nails were directed to pass through the bone cyst, one at a time. **(D) **Iliac crest bone marrow was mixed with demineralized bone matrix and the mixture was injected at the cyst.

With the child under general anesthesia without the use of a tourniquet, fluoroscopy is used to locate the cyst and the physis. After full skin preparation and draping, a thin trocar is used to perforate the bone cortex and evacuate the cyst by aspiration of fluid. A cystogram was performed; a venous drainage was not observed in any of the patients in this study. The cysts would not have been injected with bone marrow and demineralized bone matrix if a large venous drainage was observed. Using a thin curette under fluoroscopic guidance tissue was obtained for histology (Figure 2A). Histology sections showed a unicameral bone cyst in all the patients.

Under fluoroscopic guidance, retrograde intramedullary nailing was done using flexible titanium intramedullary nails; the 2 nails were ECMES nails (ECMES nail™, DePuy International Ltd., Leeds, UK), and the 7 nails were BIOMET nails (BIOMET^® ^WIN flexible nails, Biomet, Inc. Parsippany, NJ, USA). These nails are 2 to 4 millimeters in diameter and can be trimmed to the appropriate length. The diameter and length of the nails were selected on the basis of measurements made with a tape on the preoperative anteroposterior radiograph; the enlargement on radiographs was taken into account. The chosen length of the nails was then rechecked with the image intensifier after placement of the nail on the anterior surface of the corresponding bone. The longest nails that did not encroach on the proximal growth plate were used. The diameter of the nails was selected such that two nails would occupy approximately two-thirds of the medullary canal. Two straight stab wound incisions were made in the distal region of the involved part of the extremity, medially and laterally, approximately one to two centimeters proximal to the joint line. The cortical bone was exposed by blunt dissection; under image-intensifier control, k-wires were drilled in appropriate positions on both medial and lateral cortices (Figure 2B). The entry holes were created with cannulated drills over the k- wires. To avoid irritation of the ulnar nerve a small incision was made to allow the secure placement of the drill sleeve firmly on the surface of the bone. Both nails were directed to pass through the bone cyst, one at a time. The proximal and distal physes were avoided (Figure 2C). The distal ends of the nails were appropriately trimmed and left protruding from the bone without acute bending to avoid irritation of the soft tissues while ranging the adjacent joint. Autologous bone marrow was then aspirated from the iliac crest, using a wide-bore or a Jamshidi needle and a syringe. According to the literature [33], to ensure the greatest possible proportion of marrow to blood and to avoid premature clotting, a minimum of 10 ml were aspirated by multiple punctures 1 to 2 cm apart (1–2 ml bone marrow aspirate from each site). Bone marrow was mixed with demineralized bone matrix (Ignite, Wright Medical Technology, Inc., Arlington Tennessee, TN, USA) and the mixture was injected into the cyst (Figure 2D).

### Evaluation

All patients were reviewed clinically and radiographically at 2 and 6 weeks and at 3 and 12 months in the first year, at 6 months in the second year, and then annually. Clinical evaluation included the presence of pain, the participation in daily living activities, the range of motion of the adjacent joints and the occurrence of a fracture or re-fracture. Healing of the cysts was assessed on plain radiographs according to the criteria of Neer [3]. No nail has been retrieved at the time of this study.

## Results

The mean follow-up was 77 months (range, 5 to 8 years). Mean ratio of the cysts at diagnosis was 4.6 (range 3.2 to 6.3). The mean volume of demineralized bone matrix and bone marrow injected was 25 ml (range, 15 to 50 ml). The mean hospital stay of the patients was 24 hours. Patients with cysts in the lower extremities were kept non-weight-bearing on crutches for 4 weeks, and then gradually returned to full weight-bearing function.

At 6 weeks postoperatively, all patients were pain free and had full range of motion of the adjacent joints. Full activity including weight-bearing was resumed within this time in all children. Until the latest examination, 5 to 8 years after treatment, there was no clinical or radiological evidence of fracture or re-fracture and recurrence of the cysts. The only complications reported were irritation at the entry sites of the nails.

Radiographic signs of cyst healing were present at 3 months in all patients. Repeated injection of demineralized bone matrix or autologous bone grafting was not required in any of the patients.

**Figure 3:**
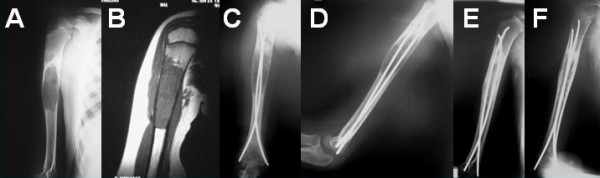
**(A) **Plain radiograph and **(B) **magnetic resonance imaging of the right humerus of a 7 year-old boy with a simple bone cyst. The patient had previous steroid injections. **(C) **Anteroposterior and **(D) **lateral radiographs after intramedullary nailing and injection of demineralized bone matrix and autologous bone marrow from the ipsilateral iliac crest. **(E) **Anteroposterior and **(F) **lateral radiographs at 31 months after the operation show complete healing of the cyst (Neer stage I).

**Figure 4:**
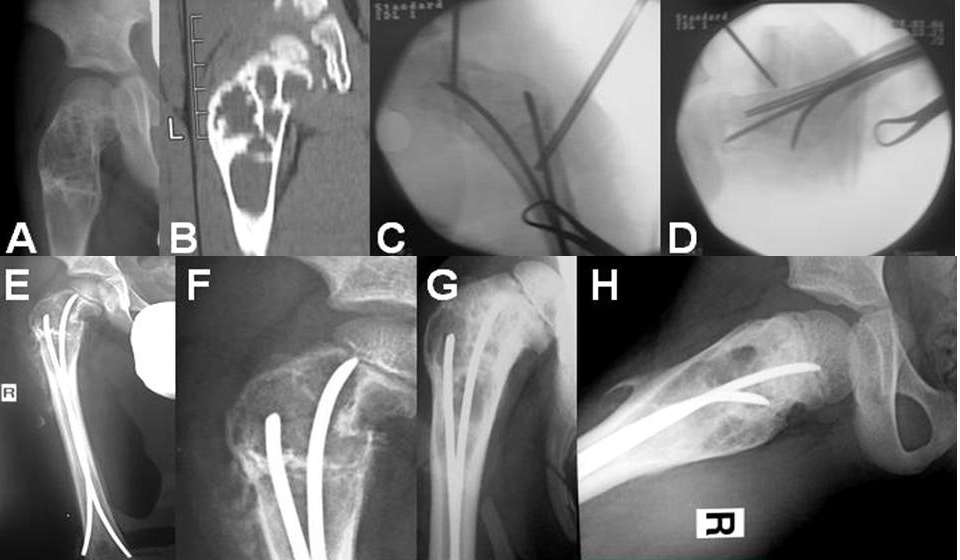
**(A) **Plain radiograph and **(B) **computed tomography scan of a 6 year-old boy with a simple bone cyst at the right proximal femur. **(C **and **D) **Intramedullary nailing and injection of demineralized bone matrix and autologous bone marrow from the ipsilateral iliac crest was done.**(E **and **F) **Plain radiographs of the right proximal femur show a subcapital femoral neck fracture after a fall from a bike three weeks postoperatively. **(G) **Anteroposterior and **(H) **lateral radiographs of the right proximal femur nine months postoperatively, show thickening of cortex and healing of the cyst (Neer stage II).

According to the classification system of Neer, there are 4 stages of cyst healing. Stage I include complete cyst filing; stage II include partial cyst filling with thickening of the cyst wall and small lucencies of less than 3 cm; stage III include recurrence of the cyst and lucencies of more than 3 cm; and stage IV include no response of the cyst [3]. In the present series, review radiographs showed that all 9 cysts had consolidated completely or partially (Neer stages I and II); 7 cysts were graded as Neer I, and 2 cysts were graded as Neer II (Figures 3 and 4).

## Discussion

Simple or unicameral bone cysts are characterized by their tenacity and their prevalence of recurrence after treatment. To some extent, this explains the diverse methods used to achieve consolidation of the cyst [1].

Initial treatment of unicameral bone cysts consisted of curettage and bone grafting [1,3,34]. However, the success rate following open procedures has ranged from 55% to 65%. The remaining 35% to 45% of patients have had recurrence of the cyst, requiring additional open surgical procedures [4,9-11,35-37]. In addition, aggressive surgical options have been related to more complications including infection, coxa vara, physeal damage, epiphyseal arrest and shortening of the limb, increased intraoperative blood loss, intraoperative fractures, and a prolonged period of postoperative immobilization [1,4,9,10,29,35,36]. Campanacci et al [1] reported 14% of retardation of longitudinal growth and limb-length discrepancy, possibly ensuing from a surgical lesion of the growth plate rather than the action of a cyst located adjacent to the physis.

As a result of the high reoperation rate and considerable morbidity associated with open surgical procedures, alternate methods of treatment have been pursued.

The treatment of simple bone cysts has significantly evolved since the percutaneous injection of methylprednisolone acetate was introduced by Scaglietti et al in 1974 [16,17]. The mechanism of action of methylprednisolone is unclear. It is postulated that the membranous wall of the cyst degenerates after the injection of corticosteroids, thus eliminating the production of fluid in the cyst and inducing the onset of osteoblastic activity [16-18]. However, long-term studies of percutaneous injection of methylprednisolone acetate have not proven the initial satisfactory results. Multiple percutaneous corticosteroid injections may be required with unpredictable results for recurrent cysts and cysts showing no response, and patients have to avoid strenuous activities for as long as the cyst heals [1,38-40].

**Figure 5:**
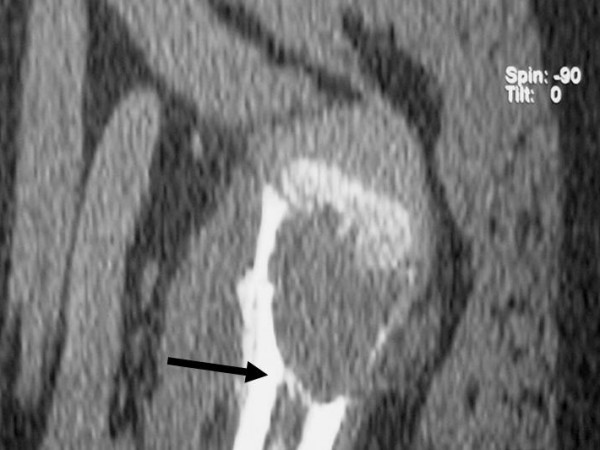
A bony plug (arrow) may cause venous obstruction and increased pressure of the interstitial fluid that may lead to the formation of a unicameral or simple bone cyst.

Several authors have advocated percutaneous multiple drilling with Kirschner wires [26,41-43], or prolonged decompression of the cyst using cannulated screws left in place without corticosteroid injection [25]. The mechanism of action of percutaneous drilling and decompression of the cysts is based on the concept that the lesions are caused by the interstitial fluid that is unable to escape from the bone because of a bony plug and venous obstruction (Figure 5). Drilling leads to a continuous decompression and decrease of the internal pressure within the cyst because of drainage of fluid through the cyst wall [25,26,41,42,44,45].

Biological methods of treatment of simple bone cysts such as autogenous or allogeneic bone grafting and injection of bone marrow or bone substitutes have also been reported [19-24]; these methods have been based on clinical studies that have suggested that aspirated bone marrow has value as a bone-graft material [46-49]. Muschler et al [33] evaluated the optimum volume of aspirated bone marrow to define the number of osteoblast progenitor cells in the aspirate. Their data indicated that, as the aspiration volume increases, the number of osteoblast progenitor cells in the aspirate increases. However, contamination by peripheral blood also increased as the aspiration volume increased, resulting in dilution of the bone marrow aspirate and decrease of the concentration of bone-marrow-derived cells. They recommended that in the setting of bone grafting, the bone marrow aspiration volume should be limited to 2 ml or less from each site in order to maximize the number of osteoblast progenitor cells in the graft site. Four 1 ml aspirates will provide almost twice the number of bone-marrow-derived cells as one 4 ml aspirate [33].

Other investigators have attempted to improve osteogenic efficiency of bone marrow by combining autologous marrow with demineralized bone matrix [50-55] and bone xenografts [56,57]. These studies showed that bone marrow graft composites produce encouraging mechanical and radiographic effects in osseous defects that could not be filled adequately by marrow alone [58].

Osteogenic elements of bone marrow or bone marrow substitutes could also promote cyst healing by stimulating bone formation [19-24]. Lokiec et al [19] reported consolidation of the cyst in all 10 patients managed with percutaneous autologous marrow grafting, in addition, however, to multiple perforations of the cyst before the injection. Delloye et al [20] proposed that simple decompression by fluid aspiration with large gauge trocars followed by a single autologous bone marrow injection promotes osteogenic healing of simple bone cysts [20]. Pimpalnerkar et al [23] reported 25% partial healing, and one major complication with autologous bone marrow injection in 32 cysts. Killian et al [21] reported healing of the cyst in 9 of 11 patients with a single percutaneous injection of demineralized bone matrix within 4–5 months, and no recurrences at 2 years follow-up [21]. Rougraff and Kling [24] reported successful results on the treatment of 23 simple bone cysts with percutaneous injection of an average of 18.7 ml (range, 5 to 32 ml) of allogeneic demineralized bone matrix and autogenous bone marrow. All patients in their series reported complete pain relief at an average of 5 weeks (range, 2 to 6 weeks), and return to full, unrestricted activities at an average of 6 weeks (range, 5 to 8 weeks). The first radiographic signs of graft incorporation were present at 3 months in all but 2 patients, and mature cortical thickening was seen in almost every patient by 1 year [24].

Although almost all the previously described methods including injection of steroids, bone marrow or bone substitutes, and decompression may produce consolidation of the cyst, they do not provide an early mechanical stability to the weakened bone. In 1981, Catier et al reported successful results of flexible intramedullary nailing for the treatment of a unicameral bone cyst in the proximal femur in 2 patients [27]. The essential feature of this method is the same as that described in earlier series that is the decompression of the cyst and the decrease in the intra-cystic pressure [26,29,30,41,44,59]. Since then, several authors have reported on intramedullary nailing of simple bone cysts as an easy, minimally invasive surgical approach that provides complete healing of the cysts, decompression and early stability to the bone, and early mobilization of the patients without any major complications [28-30,60-62].

We employed a radiographic cyst ratio for the selection of patients with large and active simple bone cysts that should be treated with combined biological and mechanical treatment using bone marrow and demineralized bone marrow injection, and cannulation-stabilization with elastic intramedullary nailing. To the best of our knowledge, the presented herein study is the only study reporting on the combined biological and mechanical treatment of large, active simple bone cysts, providing immediate mechanical stability of the lesion through a single, closed operation. Although a small series that lacks a control group, results were excellent without any major complications or need for re-operations. Radiographic evaluation showed complete or partial healing of the cysts in all patients. The time to healing was short and patients return to full daily activities without restrictions or protective splints once there was no functional pain. At the latest examination, 5 to 8 years after treatment, there was no clinical or radiological evidence of recurrence of the cysts.

## Conclusion

Combined mechanical and biological treatment of large and active simple bone cysts in long bones using demineralized bone matrix and bone marrow injection, and elastic intramedullary nailing for cyst cannulation, decompression and stabilization is effective. The proposed herein radiographic cyst ratio can be employed as an alternative to the already known cyst indexes for the selection of patients that can be treated with this treatment method.

Bone marrow or demineralized bone matrix injection alone could be performed for smaller lesions. However, the combination of fresh autologous bone marrow with biological or synthetic biocompatible material such as demineralized bone matrix that favors the induction of new bone may provide improved methods for autogenous bone grafting procedures. In addition, despite their benign nature, simple bone cysts interfere with activities of daily living. Elastic intramedullary nailing has the twofold benefits of continuous cyst decompression, and early immediate stability to the involved bone segment, which permits early mobilization and return to the normal activities of the pre-teen patients.

## Competing interests

The author(s) declare that they have no competing interests.

## Authors' contributions

ADK concieved the idea and helped in preparation of manuscript; AFM performed the literature search and helped with the draft manuscript; PJP and PNS edited the manuscript for its scientific content.

All authors read and approved the final manuscript.
